# Association of urinary bisphenol A levels with heart failure risk in U.S. adults from the NHANES (2003–2016)

**DOI:** 10.3389/fcvm.2024.1329586

**Published:** 2024-05-02

**Authors:** Yuanyuan Ma, Haobin Huang, Haiyun Qian, Yanhu Wu, Zhe Gao

**Affiliations:** ^1^ School of Public Health, Nanjing Medical University, Nanjing, China; ^2^ Department of Cardiovascular Surgery, The First Affiliated Hospital, Nanjing Medical University, Nanjing, China; ^3^ Department of Cardiothoracic Surgery, Jingzhou Central Hospital, The Second Clinical Medical College, Yangtze University, Jingzhou, China; ^4^ Department of Cardiothoracic Surgery, Children’s Hospital of Nanjing Medical University, Nanjing, China

**Keywords:** bisphenol A, heart failure, NT-proBNP, cut-off, NHANES

## Abstract

**Introduction:**

Although heart failure (HF) has been linked to bisphenol A (BPA), few studies have investigated the cut-off values for the effects of urinary BPA levels on heart failure risk. The association between urinary BPA levels and HF prognosis has not been investigated.

**Methods:**

This study included 11,849 adults over 20 years old using information from the National Health and Nutrition Examination Survey (NHANES), which was conducted from 2003 to 2016. The relationship between urinary BPA levels and the risk of HF was determined via a multivariable logistic regression model, and restricted cubic spline (RCS) methods were used to determine the cut-off for the effect of BPA levels on HF risk. Based on the available NT-proBNP concentration data from the NHANES (2003–2004), multivariable linear regression was applied to determine the linear association between the NT-proBNP concentration and urinary BPA concentration.

**Results:**

The results revealed a positive correlation between a urinary BPA concentration in the fourth quartile and the occurrence of heart failure [OR 1.49, 95% CI (1.09, 2.04), *p* = 0.012]. A one-unit increase (1 ng/mg creatinine) in the ln-transformed BPA concentration was linked to a 15% increase in the incidence of HF [OR 1.15, 95% CI (1.03, 1.29), *p* = 0.014]. The cut-off urinary BPA concentration for HF risk was 1.51 ng/mg creatinine. There was a positive correlation between urinary BPA and NT-proBNP concentrations [*β* = 0.093, 95% CI (0.014, 0.171), *p* = 0.02] in males, but there was no linear association [*β *= 0.040, 95% CI (−0.033, 0.113), *p* = 0.283] in females.

**Discussion:**

Increased urinary BPA levels are linked to an increased risk of heart failure and poor prognosis. There is a significant increase in the risk of heart failure if the urinary concentration of BPA exceeds 1.51 ng/mg creatinine.

## Introduction

As the terminal stage of a variety of cardiovascular diseases, heart failure (HF) has a high mortality rate and dismal prognosis, with a 5-year mortality rate of 45%–60% (1, 2). Available data show that HF currently affects 64 million people worldwide and results in 10 million lost DALYs (3). The 2022 AHA/CAC/HFSA guidelines emphasize that risk factors such as hypertension, atherosclerosis, diabetes, and obesity contribute to susceptibility to HF (4). In addition to the typical risk factors listed above, previous research has revealed that environmental factors are intimately related to the development of HF. For example, long- or short-term exposure to airborne particulate matter (PM_2.5_ or PM_10_) increases the risk of HF (5). Dietary exposure to polychlorinated biphenyls (PCBs) and increased urinary cadmium concentrations have been positively linked to the risk of HF (6, 7). Blood lead levels are inversely correlated with left ventricular stroke volume and ejection fraction (8) and with reduced left ventricular systolic function (9), possibly leading to HF. Therefore, identifying the environmental risk factors associated with the development of HF is critical for its prevention.

Bisphenol A (BPA) is an industrial chemical that is used in the manufacturing of various polymers and resins (10). Some European Union countries have formulated regulations to restrict BPA use. For example, France prohibits the use of BPA in food packaging for consumers of all ages. However, in Belgium, the addition of BPA to specific food contact materials is prohibited only for children. Although BPA use is restricted, BPA has still been detected in the human body in recent years due to its environmental accumulation and bioaccumulation (11, 12). Therefore, the effect of BPA on human health cannot be ignored (13, 14).

Previous research has indicated that BPA can induce a variety of harmful health effects, including endocrine disruption, cytotoxicity, and reproductive toxicity (15). A recent study indicated that bisphenol A (BPA) is associated with an increased risk of HF. Specifically, each incremental increase in the BPA level (µg/g creatinine) corresponds to a 19% increase in the likelihood of HF (16). In an animal model, BPA also caused cardiac cell hypertrophy in mice (17). This evidence suggests that BPA may cause HF. However, few studies have investigated the relationship between urinary BPA levels and HF prognosis. N-terminal pro-B-type natriuretic peptide (NT-proBNP) serves as a clinical indicator of HF prognosis. NT-proBNP is a peptide hormone discharged by ventricular cells in reaction to augmented tension within the ventricular walls (18). Manal et al. reported that carbon monoxide can increase NT-proBNP levels (19). Drug treatment for breast cancer can also cause an increase in NT-proBNP levels (20). However, there are currently no reports about the associations between BPA and NT-proBNP levels.

The general population from the NHANES was used in this study to investigate the association between urinary BPA concentrations and HF risk. Subsequently, the association between BPA and NT-proBNP concentrations was explored for the first time to investigate the link between BPA concentrations and HF prognosis. The different effects and associations in males and females were investigated by performing a stratified analysis by sex.

## Methods

### Study population

Our research data were derived from the NHANES, an ongoing cross-sectional study conducted to monitor the health of noninstitutionalized civilians in the United States since 1999. The survey includes questionnaire interviews concerning demographic, socioeconomic, dietary, and health characteristics, as well as physical examinations and laboratory tests. Serum, plasma, and urine samples are collected from the participants and processed in mobile examination centres (MECs). Additional details regarding the survey are available in the online resources of the NHANES (www.cdc.gov/nchs/nhanes.htm). The NHANES has received appropriate ethical approval, and research concerning the population aligns with ethical standards.

**Figure 1:**
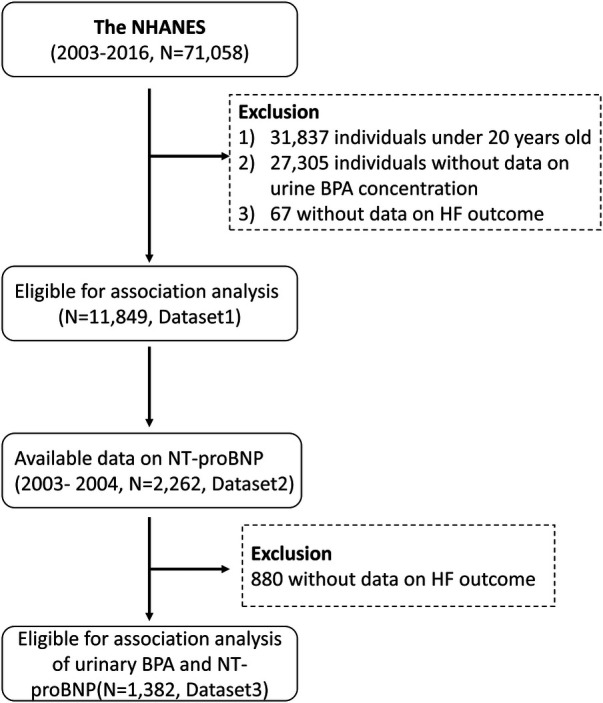
A flowchart of the data integration and analysis process.

We aggregated NHANES data spanning from 2003 to 2016, aiming to optimize the sample size to include available urinary BPA concentration and HF data. In total, 11,849 participants were included in the analysis of urinary BPA levels and HF risk after excluding 31,837 participants under 20 years old, 27,305 participants without urinary BPA concentration data and 67 participants without HF outcome data. Then, we obtained data from the year in which the BNP test data were obtained. NT-proBNP data were available for 2,262 NHANES participants from 2003 to 2004. In addition, we excluded individuals without HF outcome data (*N* = 880), and 1,382 participants were included in the association analysis of urinary BPA and NT-proBNP concentrations. A flowchart of the data integration and analysis process is shown in Figure 1.

### Assessment of outcomes

Heart failure was defined by self-reported physician diagnoses and medical status questionnaires completed during individual interviews. Participants were identified as having HF if they responded affirmatively to the structured question. Comprehensive information regarding the question is available in the Supplementary Materials. Individuals who answered that they did not know whether they had been diagnosed with congestive heart failure (CHF) were excluded from our study. The CHF outcome was converted into a dichotomous variable.

### Assessment of urinary BPA concentrations

Total (free and conjugated) urinary BPA was one of the biological variables evaluated in the NHANES. Spot urine samples were collected from participants during the interview period. Prior to testing, the urine samples were frozen and preserved on dry ice before being transported to the National Environmental Health Center. BPA concentrations were measured in urine samples from a one-third random subsample of the participants. The measurement methods have been described previously (21). Detailed measurements are available in the NHANES Laboratory Procedures Manual (22). Considering the effect of other urinary components and urinary dilution, the urinary BPA concentration was adjusted by urinary creatinine (BPA/Cr), which is a common method for assessing chemical exposure (23). When the detectable concentration of BPA was less than the lower limit of detection (LLOD) (0.4 ng/ml), the value was replaced by the square root of 2.

### Measurement of NT-proBNP

Blood samples taken during the NHANES (1999–2004) were used for NT-proBNP laboratory testing. The NT-proBNP data included in our study can be referred to as the NHANES 2003–2004 laboratory data. For detailed testing methodologies, please see the Supplementary Materials. NT-proBNP remains stable in samples subjected to up to four freeze-thaw cycles or in frozen samples previously stored at 4°C for 24 h (24). These measurement and storage conditions ensure the stability of NT-proBNP.

### Covariates

Some variables were chosen as potential confounding factors in our study, including demographic information (age, sex, race, socioeconomic status, and education level), health-related factors [body mass index (BMI), alcohol consumption, and physical activity level], and metabolite levels (serum cotinine and urinary creatinine). The serum cotinine concentration was considered an indicator of smoking status and exposure to secondhand smoke. The questionnaire used for the baseline interview yielded comprehensive information. Comprehensive information about the covariates examined in this study can be found in the Supplementary Materials.

### Statistical analysis

The baseline characteristics of the participants are reported according to the incidence of HF. Categorical variables are presented as proportions (%), whereas continuous variables are presented as the means with standard deviations or medians with interquartile ranges (IQRs). Differences in continuous and categorical variables between the groups were assessed using the Mann–Whitney *U* test and chi-squared test, respectively.

Due to the skewed distribution, the natural logarithm (ln) was used to transform urinary BPA (ng/mg, creatinine) and NT-proBNP (pg/ml) concentrations. We constructed a multivariable logistic regression model to estimate the association between urinary BPA concentrations (continuously distributed or categorized into quartiles) and HF risk after we adjusted for potential confounding variables (details of the covariates selected in this study are described in the Supplementary Material). The outcomes reveal the effect estimates (ORs) and their corresponding 95% confidence intervals (CIs) for each quartile in relation to the reference quartile (Q1 group). Furthermore, we conducted a sex-stratified analysis to explore sex differences between males and females. The RCS model was used to visualize the exposure-response relationship between urinary BPA concentrations and HF risk and the cut-off values. We used multivariate linear regression to determine the relationship between the HF prognosis biomarker (NT-proBNP concentration) and urinary BPA concentration.

Data processing and baseline characteristic analyses were carried out using STATA MP16 (SAS Institute, Cary, NC, USA), and association or mediation analysis was carried out using R 4.1.3 (R-Foundation for Statistical Computing, Vienna, Austria). Every test was two-sided, and a *p* value of 0.05 or lower was considered to indicate statistical significance.

## Results

### Baseline characteristics

Table 1 provides comprehensive details regarding the baseline characteristics of the participants. Among the 11,849 participants, 371 participants were diagnosed with heart failure. The participants’ average age was 49 ± 18 years, and the age of the population with HF outcomes was older than that of the healthy population (*p* < 0.001). A total of 48.7% of the participants were male, and the majority of the participants were non-Hispanic white. A BMI indicating obesity, an educational level less than the 11th grade, a low physical activity level, and a family history of CVD were more common in heart failure patients. The median creatinine-corrected BPA concentration was 1.56 [0.91, 2.80] (ng/mg creatinine), and individuals with HF tended to have higher urinary BPA concentrations.

**Table 1:** Baseline characteristics of the 11,866 participants by incidence of heart failure.

Baseline characters	Overall	Presence of heart failure		*p* value
		Yes	No	
No. of participants	11,849	371	11,478	
Age (years)	49.0 ± 18.0	67.0 ± 12.8	48.4 ± 17.8	<0.001
Sex (%)				<0.001
Male	48.7	58.8	48.4	
Female	51.3	41.2	51.6	
Ethnicity (%)				<0.001
Mexican American	16.2	9.7	16.4	
Other Hispanic	8.8	7.0	8.9	
Non-Hispanic White	43.9	52.3	43.6	
Non-Hispanic Black	21.5	27.5	21.3	
Other race, including multiracial	9.6	3.5	9.8	
BMI (%)				<0.001
Normal (<25 kg/m^2^)	29.5	20.8	29.7	
Overweight (25–29.9 kg/m^2^)	32.8	26.2	33.1	
Obesity (≥30 kg/m^2^)	36.6	51.2	36.2	
Missing	1.1	1.8	1.0	
Family PIR				<0.001
<1	19.5	27.0	19.3	
≥1	72.3	63.3	72.6	
Missing	8.3	9.7	8.1	
Education level (%)				<0.001
Less than 9th grade	11.2	20.8	10.9	
9–11th grade	14.9	22.6	14.6	
High school grade/GED or equivalent	23.2	22.1	23.3	
Some college or AA degree	28.4	24.3	28.5	
College graduate or above	22.2	10.2	22.6	
Missing	0.1	0	0.1	
Physical activity level				<0.001
None	54.1	65.8	53.8	
Moderate	23.9	19.9	24.0	
Vigorous	21.3	10.5	21.6	
Missing	0.7	3.8	0.6	
Diabetes				
Yes	12.6	40.7	11.7	
No	87.4	59.3	88.3	
Family history of CVD				<0.001
Yes	14.5	24.8	14.1	
No	83.0	70.4	83.4	
Missing	2.5	4.8	2.5	
Alcohol consumption (%)				0.007
<12 drinks per year	26.4	33.4	26.1	
≥12 drinks per year	65.0	58.8	65.2	
Missing	8.6	7.8	8.7	
Serum cotinine category (%)				0.28
<LOD	23.82	21.29	23.90	
LOD-10	47.33	46.09	47.37	
>10	24.18	26.42	24.11	
Missing	4.68	6.20	4.63	
Bisphenol A, ng/mg creatinine	1.56[0.91, 2.80]	1.67[0.97, 2.98]	1.56[0.90, 2.78]	0.03
Urinary creatinine (mg/dl)	126.1 ± 80.5	117.7 ± 80.8	126.3 ± 80.5	0.01
NT-proBNP (pg/ml)	191.5 ± 732.8	150.8 ± 483.0	1,214.3 ± 2,682.4	0.0001

PIR, family income-to-poverty ratio; BMI, body mass index; LOD, limit of detection.

The LOD of serum cotinine was 0.011 ng/ml.

Continuous variables are presented as the mean ± standard error or median [IQR].

### Association of urinary BPA concentrations with HF risk

**Figure 2:**
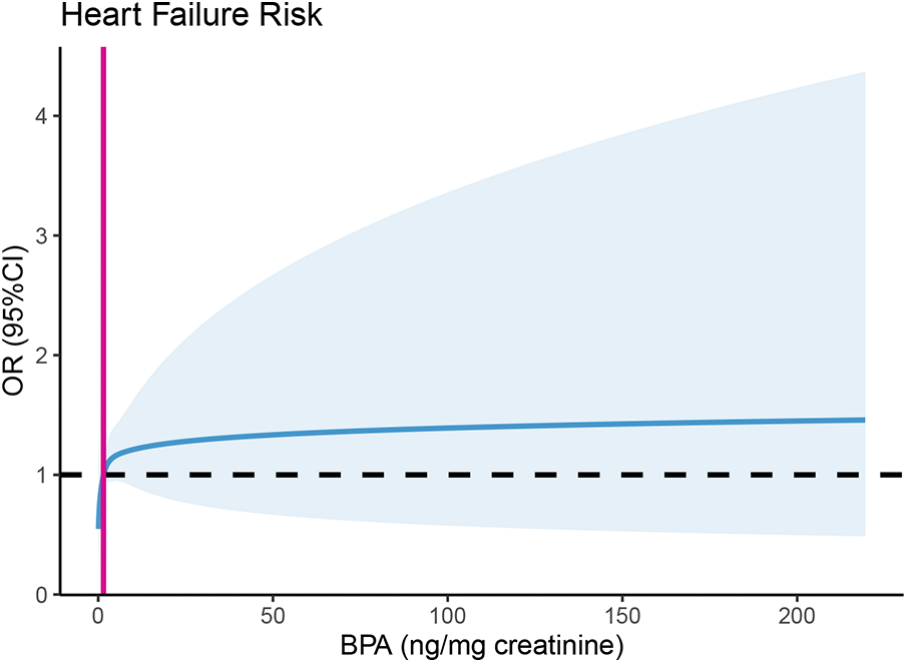
Predicted spline curves for the association between urinary BPA concentrations and heart failure risk using restricted cubic spline (RCS) regression. Restricted cubic spline regression adjusted for age, sex (male, female), race (non-Hispanic white, non-Hispanic black, Mexican American, others), education level (less than 9th grade, 9–11th grade, high school grade/GED or equivalent, some college or AA degree, college graduate or above), BMI (normal weight, overweight, obese), physical activity level (none, moderate, vigorous), PIR (<1, ≥1), alcohol consumption (<12 drinks per year, ≥12 drinks per year), and cotinine (< LOD, LOD-10, >10). NT-proBNP, N-terminal prohormone B-type natriuretic peptide; BPA/Cr, creatinine-corrected urinary BPA.

Table 2 displays the link between urinary BPA concentrations and HF risk. According to the analysis of all individuals’ quartiles, the incidence of HF was positively correlated with a urinary BPA concentration in the fourth quartile [OR 1.49, 95% CI (1.09, 2.04), *p* = 0.012]. The significant relationship between a urinary BPA concentration in any quartile and HF risk disappeared in females, while a urinary BPA concentration in the second quartile was significantly related to HF risk in males [OR 1.54, 95% CI (1.03, 2.31), *p* = 0.036]. Additionally, we assessed the relationship between continuous urinary BPA concentrations after ln-transformation and HF outcomes. According to the findings, there was a 15% increase in the incidence of HF for every one-unit (ng/mg creatinine) increase in the ln-transformed urinary BPA concentration [OR 1.15, 95% CI (1.03, 1.29), *p* = 0.014]. A sex-specific stratified analysis revealed that although the aforementioned association disappeared in males, the ln-transformed BPA concentration was positively associated with HF risk in females [OR 1.20, 95% CI (1.09, 1.43), *p* = 0.044]. As shown in Figure 2, we used restricted cubic splines to visualize the relationship between urinary BPA concentrations and HF risk. The risk of HF was relatively stable until a urinary BPA/Cr value of approximately 1.51 ng/mg was reached and then started to increase afterwards. Beyond this point, the risk of HF increased, reaching 9.91 ng/mg, after which it plateaued and gradually increased.

**Table 2:** Association between urinary bisphenol A (BPA) levels and CVD risk among U.S. adults, NHANES 2003–2016.

BPA	Congestive heart failure		
	OR^a^	95% CI	*P* value
Total participants			
Quartile 1	Reference		
Quartile 2	1.24	(0.90, 1.71)	0.186
Quartile 3	1.37	(1.00, 1.88)	0.052
Quartile 4	1.49	(1.09, 2.04)	0.012*
Ln-transformed BPA	1.15	(1.03, 1.29)	0.014*
Male			
Quartile 1	Reference		
Quartile 2	1.54	(1.03, 2.31)	0.036*
Quartile 3	1.46	(0.96, 2.22)	0.074
Quartile 4	1.47	(0.97, 2.22)	0.069
Ln-transformed BPA	1.12	(0.97, 1.30)	0.129
Female			
Quartile 1	Reference		
Quartile 2	0.83	(0.48, 1.43)	0.504
Quartile 3	1.22	(0.74, 2.01)	0.429
Quartile 4	1.46	(0.90, 2.36)	0.127
Ln-transformed BPA	1.20	(1.09, 1.43)	0.044*

NHANES, National Health and Nutrition Examination Survey; BPA, bisphenol A (ng/mg creatinine).

^a^ Logistic regression adjusted for age, sex (male, female), race (non-Hispanic white, non-Hispanic black, Mexican American, others), education level (less than 9th grade, 9–11th grade, high school grade/GED or equivalent, some college or AA degree, college graduate or above), BMI (normal weight, overweight, obese), physical activity (never, moderate, vigorous), PIR (<1, ≥1), alcohol consumption (<12 drinks per year, ≥12 drinks per year), and cotinine (<LOD, LOD-10, >10), and urine creatinine.

* *p* < 0.05.

### Association of urinary BPA concentrations with NT-proBNP concentrations

The multivariate linear regression results showed that the concentration of NT-proBNP was positively related to the concentration of urinary BPA [*β *=* *0.053, 95% CI (0.006, 0.099), *p* = 0.028]. A positive association between urinary BPA and NT-proBNP concentrations was detected in males [*β *= 0.093, 95% CI (0.014, 0.171), *p* = 0.02], while no linear association between BPA and NT-proBNP concentrations was detected in females [*β *=* *0.040, 95% CI (−0.033, 0.113), *p* = 0.283].

## Discussion

This study revealed that BPA concentrations are associated with HF risk, and the cut-off urinary BPA concentration for increased HF risk was above 1.51 ng/mg creatinine. BPA concentrations were also shown to be associated with NT-proBNP concentrations for the first time, highlighting the poor prognosis of heart failure patients with BPA exposure.

Although previous studies have demonstrated that BPA exposure is associated with several cardiovascular disease (CVD) outcomes (25), such as myocardial infarction (MI), coronary heart disease (CHD) and hypertension, there are currently few reports on the associations between BPA and HF. The results of a meta-analysis incorporating multiple epidemics also revealed an association between an increased urinary BPA concentration (adjusted by urinary creatinine) and an increased risk of HF (23). In addition, compared to the highest and lowest levels of exposure to the BPA substitute bisphenol F, exposure to bisphenol F increased the risk of HF by 15% (26). According to the findings in the present study, the risk of HF increased by 15% for every one-unit (ng/mg creatinine) increase in the ln BPA/Cr ratio, which is consistent with previous findings. However, the association between urinary BPA concentrations and HF risk differed according to sex. In contrast to the findings of Li et al. (16), the ln-transformed BPA/Cr ratio was positively associated with heart failure risk in females, while the aforementioned association disappeared in males. This opposite conclusion may be due to the varying covariates used in the logistic regression model. In addition, the included studies were cross-sectional studies, which may have led to bias, and subsequent cohort studies are needed to verify the causal relationship. In addition, the nonlinear association between urinary BPA concentrations and heart failure risk may be because BPA, as an environmental pollutant, has a toxic effect threshold at which heart failure risk increases. The threshold for urinary BPA, below which there is no heightened risk of heart failure, was determined to be 1.51 ng/mg creatinine—a value closely approximating the median urinary BPA level within this study population. The plateau in the increased risk of heart failure is potentially associated with direct myocardial injury induced by BPA beyond a urinary concentration of 9.91 ng/mg creatinine.

At present, although there are reports of an association between BPA and heart failure, these cross-sectional studies cannot confirm a causal relationship. A mechanistic study revealed that BPA exposure may cause HF. The expression of mast cell markers increased significantly in mice treated with 25 μg/L BPA in drinking water compared to those treated with 0 μg/L BPA (17). Other mechanistic studies have shown that mast cells promote heart remodelling and fibrosis (27, 28). This evidence suggested that BPA may cause cardiac remodelling and fibrosis and further increase HF risk. Another study revealed that exposure to BPA led to prolonged PR segments and decreased epicardial conduction velocity in the heart in mice (29). After exposure to BPA, the vitality of heart cells decreases (30). This experimental evidence suggests that BPA can cause cardiac dysfunction, which may lead to HF. The sex specificity of BPA exposure and HF risk was reported in several previous animal studies. Yan et al. (31) revealed a more evident oestrogen-like effect of BPA in female mice, but no observable response was found in male mice. More epidemiological and experimental research is needed to explore the sex-specific impact of BPA exposure on the incidence of heart failure.

An elevated NT-proBNP level provides evidence for the prognosis of HF patients. In this study, we found that BPA was associated with HF and increased NT-proBNP levels, which indicated a poor prognosis for patients with HF. The sex specificity of the association between urinary BPA and NT-proBNP concentrations was reported for the first time, in which a significant association was observed for males but not for females. However, sex differences in this association need to be further investigated. This research approach may provide a possible paradigm for studying exposure to environmental chemicals and HF incidence. Many studies have shown an association between chemical treatment and HF (32–35). However, there has been no simultaneous research on the association between these environmental chemicals and NT-proBNP concentrations. Therefore, this is also the first study to analyse the relationships between exposure to environmental chemicals and NT-proBNP concentrations and HF risk.

This study has the following strengths. First, for the first time, we reported an association between urinary BPA and NT-proBNP concentrations, and similar to the findings of previous studies, we also observed an association between urinary BPA concentrations and HF risk. The positive correlation observed between urinary bisphenol A (BPA) and NT-proBNP concentrations underscores the link between urinary BPA concentrations and the risk of heart failure. Second, we identified the cut-off urinary BPA concentration, indicating no heightened risk of heart failure.

However, this study also has several limitations. First, because this study was cross-sectional in nature, it was impossible to determine whether an increased BPA concentration causes HF or whether HF causes an increase in the urinary BPA concentration. In addition, there is a lack of strong evidence for an association between urinary BPA and NT-proBNP concentrations, and additional epidemiological and experimental studies are needed to verify this conclusion. We summarized the mechanistic studies previously reported in the Discussion section. Second, several factors, such as genetic factors, were not considered. Due to the presence of genetic factors, some people are susceptible to HF. However, due to the lack of complete genetic factor information in the NHANES, we could not perform covariate correction to reduce this bias, which needs to be addressed in future research. Third, other potential biomarkers of HF were not included in the present study, mainly because of the unavailability of related data. The role of other heart markers could be investigated in future research once the data are released by the NHANES. Finally, the exposure level of BPA may change dynamically, possibly due to changes in exposure sources. This study used the urinary BPA concentration to represent a possible bias in BPA exposure. In future research, additional time points are needed to represent stable BPA exposure.

## Conclusion

Our study revealed the relationship between urinary BPA concentrations and HF risk. The cut-off BPA concentration for HF risk was 1.51 ng/mg creatinine. Urinary BPA concentrations were associated with increased plasma NT-proBNP concentrations, indicating a poor prognosis in HF patients.

## Data Availability

The original contributions presented in the study are included in the article/**Supplementary Material**, further inquiries can be directed to the corresponding authors.
