# Supplementary material for: Association of urinary bisphenol A levels with heart failure risk in U.S. adults from the NHANES (2003–2016)
Source: Front Cardiovasc Med. 2024 May 2;11:1329586. doi: 10.3389/fcvm.2024.1329586 (PMC11099872; doi:10.3389/fcvm.2024.1329586)
Supplement: Supplementary file 1 [file Datasheet1.docx]

**Supplementary material**

**Methods**

**Assessment of outcomes**

The participants were considered to suffer from heart failure if they answered “yes” to the following structured question: “Has a doctor or other health professional ever told you that you had congestive heart failure (CHF)?”.

**Measurement of NT-proBNP**

The detection was conducted at the University of Maryland School of Medicine during 2018-2020. The NT-proBNP measurement in serum was performed on the Roche Cobas e601 autoanalyzer (Roche Diagnostics) with a detection limit of 5 pg/ml–35000 pg/ml. The coefficient of variation was 3.1% at low NT-proBNP levels (46 pg/mL) and 2.7% at high NT-proBNP levels (32,805 pg/mL).

**Covariate**

The potential confounding variables were selected in our study as follows: demographic data, including age (years), sex (male/female), race (Mexican American, other Hispanic, non-Hispanic white, non-Hispanic black, and other), family income-to-poverty ratio (PIR) (<1, ≥1), educational level (less than 9th grade, 9−11th grade, high school grade/GED or equivalent, some college or AA degree, college graduate or above); health-related factors, including body mass index (BMI) (<25 kg/m2, 25 to 29.9 kg/m2, ≥30 kg/m2), alcohol consumption (<12 drinks per year, ≥12 drinks per year) and physical activity (never, moderate, vigorous); and metabolite levels, including serum cotinine (< LOD, LOD-10, >10), and urine creatinine (mg/dl). Detailed information was obtained from the baseline interview questionnaire. The PIR is a measure of socioeconomic standing, which represents family income defined in accordance with an appropriate poverty threshold [24847661]. BMI (kg/m2) was calculated by dividing weight in kilograms by the square of height in meters. Serum cotinine was determined by isotope-dilution high-performance liquid chromatography/atmospheric pressure chemical ionization tandem mass spectrometry (ID HPLC-APCI MS/MS).

**Ethics**

The NHANES received ethical approval from the National Center for Health Statistics Research Ethics Review Board, and all participants furnishing written informed consent.
